# The development of machine learning in lung surgery: A narrative review

**DOI:** 10.3389/fsurg.2022.914903

**Published:** 2022-09-12

**Authors:** Anas Taha, Dominik Valentin Flury, Bassey Enodien, Stephanie Taha-Mehlitz, Ralph A. Schmid

**Affiliations:** ^1^ Department of Biomedical Engineering, Faculty of Medicine, University of Basel, Allschwil, Switzerland; ^2^ Department of Thoracic Surgery, Hirslanden Clinic Beau-Site (Hirslanden Group) / Lindenhof Hospital (Lindenhof Group Bern); University of Bern, Bern, Switzerland; ^3^ Department of Surgery, Wetzikon Hospital, Wetzikon, Switzerland; ^4^ Clarunis, University Centre for Gastrointestinal and Liver Diseases, St. Clara Hospital and University Hospital Basel, Basel, Switzerland; ^5^ Thorax-Schweiz, Hirslanden Cooperate Office, Glattpark, Switzerland

**Keywords:** machine learning, thoracic surgery, lung surgery, deep learning, narrative review

## Abstract

**Background:**

Machine learning reflects an artificial intelligence that allows applications to improve their accuracy to predict outcomes, eliminating the need to conduct explicit programming on them. The medical field has increased its focus on establishing tools for integrating machine learning algorithms in laboratory and clinical settings. Despite their importance, their incorporation is minimal in the medical sector yet. The primary goal of this study is to review the development of machine learning in the field of thoracic surgery, especially lung surgery.

**Methods:**

This article used the Preferred Reporting Items for Systematic and Meta-analyses (PRISMA). The sources used to gather data are the PubMed, Cochrane, and CINAHL databases and the Google Scholar search engine.

**Results:**

The study included 19 articles, where ten concentrated on the application of machine learning in especially lung surgery, six focused on the benefits and limitations of machine learning algorithms in lung surgery, and three provided an overview of the future of machine learning in lung surgery.

**Conclusion:**

The outcome of this study indicates that the field of lung surgery has attempted to integrate machine learning algorithms. However, the implementation rate is low, owing to the newness of the concept and the various challenges it encompasses. Also, this study reveals the absence of sufficient literature discussing the application of machine learning in lung surgery. The necessity for future research on the topic area remains evident.

## Introduction

### Rationale

Machine learning, popularly abbreviated as ML, represents a form of artificial intelligence that permits software apps to increase their accuracy when predicting outcomes through learning while analysing data without undergoing explicit programming to perform the task. ML’s success is primarily based on its significant improvements through processing data, particularly in the image recognition sector (1), where it can learn associations with multiple characteristics of a picture and a diagnosis (training data), hence, allowing to predict a diagnosis based on an image never seen before. Machine learning classification often depends on how a specific algorithm learns to attain accuracy in its forecasts. The four standard machine learning models include supervised, unsupervised, semi-supervised, and reinforcement learning approaches. The choice of an algorithm significantly depends on the form of data used and the outcome researchers wish to predict. The application of machine learning has been substantial, e.g., with its most prominent usage being the recommendation algorithm that determines the news feed (outcome) on Facebook according to your previous search history (training data). Individuals have also used machine learning in customer relationship management, business intelligence, self-driving vehicles, and virtual assistants. The main benefit of ML is that when an algorithm learns what to do with specific information, it begins to function automatically (2). Further, it enhances businesses’ competitive edge by enabling them to view consumer trends and improve enterprises’ operational patterns.

In recent years, the medical domain has witnessed extensive attention toward championing the adoption of machine learning algorithms to improve various processes in the laboratory and clinical setting. For example, lab technicians have adopted ML to reconstruct diseases by allowing pharmaceutical companies to feign drug response at the patient level. Also, medical scientists use machine learning algorithms to facilitate hypothesis testing by enabling lab technicians to establish a hypothesis, model it, tune it, and replicate the procedure iteratively. In the clinical setting, ML ensures the development of diagnostics and the improvement of prognostics. Rajkomar, Dean, and Kohane argued that despite the importance of machine learning technology in medicine, there was very little evidence of its application in the area as of 2019 (3).

**Figure 1:**
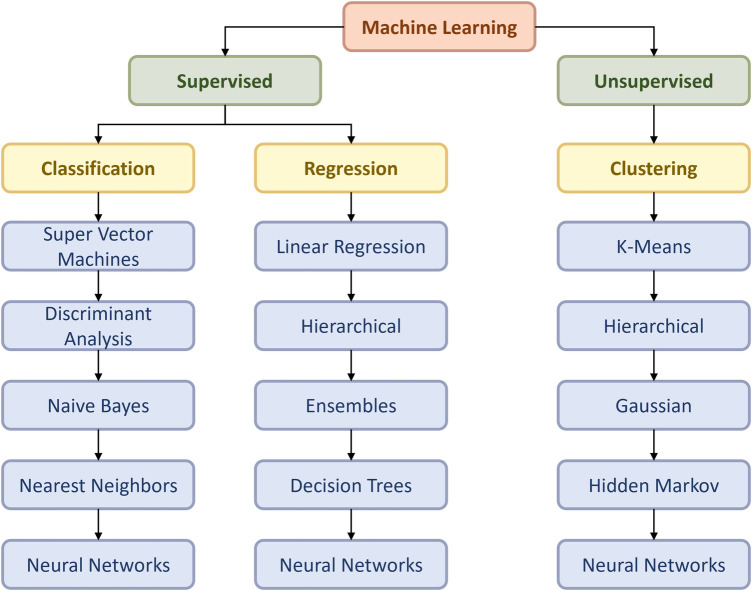
Flow chart of the ML model according to Duc TL et al. (4).

At that juncture, the main aim of this article is to review the development of machine learning in thoracic surgery, with a specific focus on lung surgery, excluding cardiac surgery. This topic is essential as it will help to identify the current status of machine learning in lung surgery, educating the public on the recent progress and future expectations. Also, the outcome of this study will help technicians in the ML field to gather information on how they can contribute to the improvement of the present algorithms in lung surgery and even the whole medical domain. The flow chart (Figure 1) below relates to the architectural structure of creation and validation of the machine learning model.

We will conduct a narrative review to acquire vital information on the development of machine learning in lung surgery. We selected this approach since it facilitated mapping out the relevant literature on a topic and evaluating the relevant concepts for informing practice. Adopting the narrative review approach to answer the research question is suitable since machine learning is a relatively new notion in surgery, given its dismal application in the entire medical domain. Borella et al. claimed that narrative reviews allow researchers to acquire a comprehensive perspective concerning different topics under evaluation (5). The other reasons for selecting this model stem from its ability to evaluate the evidence presented and the methodologies contained in the included studies. On the contrary, Pae argued that the main shortcoming of narrative reviews is that the absence of the systematic selection of studies could trigger bias during result analysis (6). Nevertheless, a narrative review will ensure the generation of a good overview of the evidence concerning machine learning in lung surgery.

### Objectives

The research question for this study is:

How has machine learning developed in the lung surgery domain?

The following research objectives will help in responding to the research question:

• To evaluate the current state of machine learning in lung surgery.
• To assess the benefits and limitations of machine learning integration into lung surgery.
• To provide an overview of the future of machine learning in lung surgery.

## Methods

### Protocol and registration

The protocol assimilated into this study was the Preferred Reporting Items for Systematic and Meta-analyses (PRISMA). The approach consists of twenty compulsory elements and two optional features that guide researchers in conducting reviews and the appropriate directions. The suitability of this methodology stems from its ability to provide comprehensive analyses of the benefits and shortcomings of studies. Further, the approach permits the evaluation of the quality of reviews, hence, ensuring the validity of derived outcomes. Page and Moher claimed that the PRISMA method had allowed researchers to conduct quality meta-analyses with the potential of informing practice (7). Other than that, the PRISMA protocol will provide opportunities for the duplication of the review methods by future researchers, hence, facilitating the gathering of quality results. Thus, this protocol will allow the researcher to detail the steps taken to derive their results.

### Eligibility criteria

Three factors determined the eligibility criteria for this study. Considering relevancy of the publications, the studies have been evaluated by their focus area. The studies had to concentrate specifically on machine learning in lung surgery to be included. We evaluated the language of the articles, whereby they excluded any publications in any other language except English. This factor ensured that they did not spend extensive time translating articles from other languages. Finally, we examined the publication dates of the articles and included the papers released from January 2015 to March 2022. This element ensured the gathering of detailed information on the subject area.

### Information sources

We conducted a literature review through databases and search engines to gather the relevant data for this study. The databases adopted entailed PubMed, Cochrane, and CINAHL. The search engine incorporated was Google Scholar. The literature search occurred on Mar 31, 2022, and the authors sought to include articles released from January 2015 to March 2022.

### Search

We integrated the search strategy of inputting key terms in the adopted databases and search engines. On the PubMed, Cochrane, and CINAHL avenues, the key terms used comprised «machine learning», «lung surgery», «deep learning», and «machine learning algorithms». When gathering information from PubMed, the researchers used the best match and 2015 to 2022 filters, while the date and trials filter was the most relevant for the Cochrane database. On CINAHL, the filter adopted was publication year to ensure that the acquired articles lie in a particular time frame.

On the Google Scholar search engine, the key phrases used to gather relevant information were «machine learning in lung surgery», and «the future of machine learning in lung surgery». Consequently, the filters incorporated on Google Scholar consisted of «January 2015 to March 2022», «sort by relevance», and «any type». Our librarian who had drafted the search strategy spearheaded the search process to ensure the smooth running of things and the gathering of relevant studies. Moreover, all the authors in the study participated in peer-reviewing the search strategy to gauge its accuracy and validity. The researchers adopted the Peer Review of Electronic Search Strategies PRESS checklist to conduct the review. According to Rethlefsen et al., this checklist ensures an evaluation of the mechanism and the thoroughness of the search strategy to guarantee quality (8). Thus, this approach was vital in ensuring that the search strategy considered the key terms of the research topic to guarantee the gathering of relevant sources.

### Selection of evidence sources

We worked in pairs to screen the acquired documents and ensure they contained the relevant data. We also examined the language used in the publications and excluded articles in other languages apart from English during this process. We further evaluated the abstracts to determine whether full texts of the acquired articles were available and excluded any abstracts without full texts. The exclusion of these articles was necessary since they could not provide definitive conclusions on the study topic. We resolved any inconsistencies by redoing the screening to derive conclusive decisions.

### Data charting

The data charting process established a form for inputting information. The form comprised of the relevant features to look for when gathering data. For instance, the form detailed factors like machine learning and its current application in lung surgery. Also, the form highlighted elements like the benefits, shortcomings, and future expectations of ML in lung surgery.

### Data items

The data items considered for the study relied on the context of the articles. We ensured that the papers concentrated on lung surgery and machine learning for inclusion.

### Synthesis

The grouping of included studies significantly depended on the issues addressed by the papers. For example, we categorized the articles into the ones focusing on the current state of ML in lung surgery, the advantages and shortcomings of ML in lung surgery, and the overview of the future of ML in lung surgery.

## Results

### Selection of evidence sources

**Figure 2:**
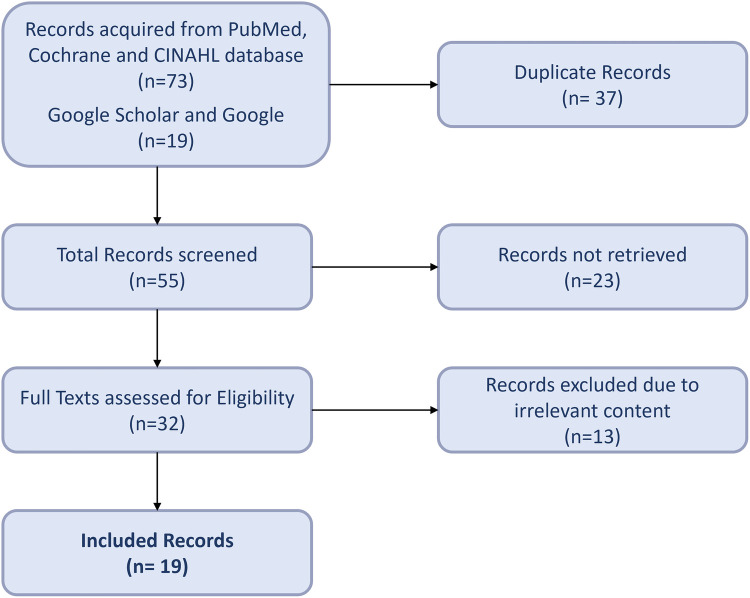
PRISMA flow diagram.

The search results from the PubMed, Cochrane, and CINAHL databases led to seventy-three results, whereas the Google Scholar search engine yielded nineteen outcomes. After removal of duplicates, fifty-five articles underwent screening. Out of the fifty-five records screened, twenty-three were not retrieved as they only contained abstracts with no full text accessible. Conclusively, the researchers included nineteen publications in the study, as displayed in Flow Chart below (Figure 2).

### Results synthesis

Out of the 19 included articles displayed in Table 1, ten concentrated on the application of machine learning in lung surgery, six focused on the benefits and limitations of machine learning algorithms in lung surgery, and three provided an overview of the future of machine learning in lung surgery.

**Table 1:** Summary of included articles.

Author	Objectives	ML algorithm	Application	Main findings
Salati et al. (9)	To test the performance of an ML model in predicting complications	XGBOOST	Predicting complications after lung resection	XGBOOST ML algorithm has the potential to predict complications after lung resection.
Desuky and El-Bakrawy (10)	To test the performance of various ML algorithms in their original and boosted formats	Naïve Bayes, Simple logistic, Multiple Perceptron, and J48	Predicting-post operative life expectancy	Naïve Bayes, Simple logistic, Multiple Perceptron, and J48 ML algorithms had a higher predictive capability in their original form than when they had undergone boosting.
Ravichandran et al. (11)	To test the prediction capability of a ML model	Deep Neural Network	Predicting post-operative life expectancy among lung cancer	A Deep Neural Network-based algorithm accurately predicted lung cancer patients’ life expectancy post-thoracic surgery.
Danjuma (12)	To compare the performance of three ML models	Multiple Perceptron	Predicting post-operative life expectancy after lung surgery	Multiple Perceptron was the most accurate ML algorithm for predicting the life expectancy of patients after lung cancer surgery. The second was J48, followed by Naïve Bayes.
Chen et al. (13)	To evaluate the performance of a ML model	CT-based radiomics	Predicting the presence of tumors among patients	CT-based radiomics predicted the presence of tumor spread *via* air spaces in patients with Stage I lung adenocarcinoma.
Wang et al. (14)	To test the predictive capability of a machine learning approach	Decision Tree	To predict the lung cancer patients in need of local treatment for pain reduction	The decision tree algorithm effectively determined if lung cancer patients required local treatment for reducing pain.
Oh et al. (15)	To examine the performance of an ML model in prediction	Computed Tomography	To predict the presence of pathological femoral cracks within lung cancer patients	Computed tomography was effective in predicting pathological femoral cracks among lung cancer patients.
Valdes et al. (16)	To compare the performance of four ML approaches	Decision Trees, Random Forests, and RUSBoost	To predict pneumonitis among lung-cancer patients	Decision Trees, Random Forests, and RUSBoost effectively predicted pneumonitis among patients with Stage I non-small lung cancer, but their accuracy mostly depends on the number of included participants.
Chang et al. (17)	To construct a ML method for predicting patient outcomes	Naïve Bayes Classifier	To predict patient outcomes after undergoing lung resection surgery	Naïve Bayes Classifier ML algorithm had the best testing results for determining patient outcomes after a lung resection surgery.
Haam et al. (18)	To evaluate the performance of three ML algorithms	Naïve Bayes, neural network, random forest, and support vector machine	To predict brain metastasis among lung adenocarcinoma patients	Naïve Bayes, neural network, random forest, and support vector machine successfully predicted brain metastasis (BM) among patients with lung adenocarcinoma after undergoing surgery through gene expression profiling.
Chong et al. (19)	To propose a deep learning model for enhancing prediction	random forest classifier	To test the predictive capability and cost efficiency of the suggested method	Cost efficiency and high prediction power of random forest classifier
Wu et al. (20)	To train an RF model for recognising conditions among patients	random forest classifier	To recognise lymph node metastasis among lung cancer patients	RFC machine learning algorithm was the best predictive model and facilitated the recognition of lymph node metastasis among patients with early T-stage non-small cell lung cancer before undergoing an operation.
Liu et al. (21)	To test the performance of an ML model in making predictions	CT-derived radiomic	To predict post-surgical progression-free survival of patients with lung adenocarcinoma	ML algorithms had the potential to assist in personalizing treatment decisions
Liang et al. (22)	To develop an approach for enhanced detection	ML-assisted deep methylation sequencing	To detect tumor-derived signals among patients with surgery-resectable lung cancer	ML algorithms improved lung cancer screening and better assessment of treatment efficiency.
Feng et al. (23)	To evaluate the diagnostic performance of an ML model	Quantitative texture analysis of CT images	To differentiate angiomyolipoma without visible fat from renal cell carcinoma	ML applications lack sufficient supporting literature.
Rabbani et al. (24)	To examine the performance of ML algorithms	Radiomics	To predict care of patients with nonsmall cell lung cancer	ML-based studies use small datasets, increasing bias chances.
Vinod and Hau (25)	Evaluate the performance of an ML-based algorithm	Radiotherapy	To test its predictive capability among lung cancer patients	The radiotherapy domain for dealing with lung cancer could benefit from incorporating ML algorithms due to their low cost
Kieu et al. (26)	To examine the performance of an ML algorithm in detection	Deep learning	To detect lung disease	ML algorithms have a chance of improving if datasets can be available for the public and the adoption of cloud computing
Hartgerink et al. (27)	To evaluate the performance of an ML algorithm	Whole Brain Radiotherapy	To examine life expectancy of patients	Machine learning algorithms have the potential for enhancing lung cancer patients’ outcomes by allowing the combination of multiple therapies

## Discussion

### Summary of evidence

#### The current state of ML in lung surgery

The integration of machine learning algorithms in lung surgery has been extensive. The incorporation has been in decision making for diagnoses, management of pulmonary diseases, assessing preoperative risks, surgical planning, and predicting treatment outcomes. For instance, a study by Salati et al. applied an ML algorithm known as XGBOOST to predict pulmonary complications in 1360 patients who had undergone lobectomy, segmentectomy, bilobectomy, and pneumonectomy lung resections (9). The ML algorithm adopted demonstrated success in predicting complications among patients included in the study. The model’s predictive ability showcases that it can enhance counseling and the perioperative management of patients who undergo lung resection (9). Another study by Desuky and El-Bakrawy provided a different argument by insisting that the predictive capacity of ML algorithms such as Naïve Bayes, Simple logistic, Multiple Perceptron, SVM and J48 was higher in the original versions than the boosted types of the five ML algorithms (boosted Naïve Bayes, boosted simple logistic, boosted multiple perceptron, boosted SVM, and boosted J48) (10). This argument implies that when predicting the life expectancy of lung cancer patients after undergoing lung surgery, scientists should introduce attribute selection and ML techniques to enhance predictive capability rather than integrating boosting since the original version ensures more accuracy.

In another study, Ravichandran et al. claimed that the adoption of machine learning algorithms like the Deep Neural Network-based approach developed by the authors demonstrated significant performance in predicting the life expectancy of lung cancer patients after undergoing lung surgery (11). Moreover, Danjuma aimed to evaluate the most accurate ML technique for predicting post-operative life expectancy of lung cancer patients after an operation by comparing three algorithms (Multiple Perceptron, J48, and Naïve Bayes) (12). The results indicated that the Multiple Perceptron technique had the highest classification accuracy of 82.3%, whereas J48 scored 81.8%. Naïve Bayes had the lowest accuracy score of 74.4%, but the author insisted that an algorithm’s quality and performance significantly rely on the clinical miner’s ingenuity (12). Therefore, these outcomes indicate that professionals in lung surgery should offer training to current and new surgeons on how to use ML techniques in their practice. Another research by Chen et al. found out that CT-based radiomics had the potential to predict tumor spread through air spaces in Stage I lung adenocarcinoma using machine learning (13). The machine algorithm adopted was the PyRadiomics technique. The approach showcased success in the preoperative prediction of tumors, which may aid surgeons in deriving the appropriate decisions before undertaking lung surgery.

Another study by Wang et al. indicated that the decision tree ML algorithm comprising VAS, bone metastases character, Frankel classification, Mirels’ score, age, driver gene, aldehyde dehydrogenase 2, and enolase one expression was effective in predicting whether lung cancer patients with bone metastases required local treatment for pain reduction (14). Further research by Oh et al. showcased that computed tomography in combination with machine learning classifiers like AdaBoost, support vector machine (SVM), gradient boosting (GB), linear discriminant analysis (LDA), and random forest showcased superior capabilities in predicting pathological femoral cracks among lung cancer patients (15). The authors concluded that machine learning algorithms could assist lung surgeons in anticipating various multifactorial challenges they face in the work environment (15). Earlier research conducted by Valdes et al. evaluated the performance of Decision Trees, Random Forests, and RUSBoost ML algorithms in predicting the presence of pneumonitis in patients with Stage I non-small cell lung cancer (16). The results implied that the accuracy of machine learning algorithms significantly depends on the number of patients included in the research and not the characteristics acquired or complexities of ML techniques (16).

More importantly, Chang et al. aimed to evaluate the performance of seven unsupervised machine learning algorithms in predicting the weaning from ventilators among patients who have just undergone lung resection surgery (17). The study outcomes indicated that the Naïve Bayes Classifier algorithm showcased the best testing results. Thus, the researchers used the approach to develop an application for examining risk based on patients’ medical information, facilitating the better prediction of patient outcomes after surgery (17). Haam et al. adopted four machine learning algorithms (Naïve Bayes, neural network (NN), random forest, and support vector machine) to determine brain metastasis (BM) among patients with lung adenocarcinoma after undergoing surgery through gene expression profiling (18). The outcomes indicated that the gene expression signatures successfully predicted BM. The arguments highlighted above reveal that the lung surgery domain, particularly lung surgery, has attempted to integrate machine learning into the sector. The most prominent use of the concept is in the prediction of patient outcomes, despite its numerous advantages in other areas such as diagnosis and surgical planning.

#### Benefits and limitations of ML in lung surgery

The integration of machine learning in lung surgery has numerous benefits, including enhancing predictability that facilitates the derivation of more appropriate decisions. A study conducted by Chong et al. revealed this benefit since the random forest classifier (RFC) ML model applied was cost-efficient (19). It also prompted the prediction of lymph node metastasis during or after surgery for early-stage adenocarcinomas with a sensitivity and specificity of 87.5 and 82.2 percent, respectively (19). In a similar perspective, Wu et al. indicated that the RFC machine learning algorithm was the best predictive model and facilitated the recognition of lymph node metastasis among patients with early T-stage non-small cell lung cancer before undergoing an operation (20). Another study by Liu et al. revealed that machine learning algorithms had the potential to assist in personalizing treatment decisions (21). This conclusion arose from the excellent performance of the nomogram comprising CT-derived radiomic features and risk factors developed by the authors in predicting post-surgical progression-free survival of patients with lung adenocarcinoma (21). Additionally, Liang et al. associated machine learning algorithms with improved lung cancer screening and better assessment of treatment efficiency after the ML-assisted deep methylation sequencing detected tumor-derived signals among patients with surgery-resectable lung cancer (22). These arguments imply that the advantages derived from application of ML in lung surgery ensure accurate predictability and opportunities for enhancing patient outcomes.

On the contrary, the main shortcoming of machine learning in lung surgery is the lack of sufficient research on using them appropriately. For instance, a study by Feng et al. revealed that most ML techniques require adopting different protocols and settings that require complex analysis in literature to avoid any errors (23). The second limitation of ML algorithms in lung surgery is the tendency of most outcome prediction researchers to adopt a small dataset that increases the chances of biased study results (24). Therefore, it remains vital to deal with the general challenges facing ML implementation to ensure the successful integration of the concept into lung surgery.

#### Overview of the future of ML in lung surgery

Various studies have examined the future direction of machine learning in lung therapy. For example, a study by Vinod and Hau revealed that the radiotherapy domain for dealing with lung cancer could benefit from incorporating ML algorithms due to their low cost (25). Also, ML techniques can ensure improved patient outcomes by enhancing the decision-making process of clinicians. In another study, Kieu et al. posited that the future of ML algorithms in lung surgery is bright, provided that the public can access datasets and hospitals adopt cloud computing processes (26). From a distinct perspective, Hartgerink et al. claimed that machine learning algorithms have the potential for enhancing lung cancer patients’ outcomes by allowing the combination of multiple therapies such as stereotactic radiosurgery with immunotherapy, shared decision-making, and personal isotoxic dose recommendation (27). At that juncture, the arguments presented above imply that the future applications of machine learning algorithms in lung surgery will significantly depend on how practitioners deal with the system’s current challenges. The advantages of machine learning will prompt its adoption since they make work easier at a low cost and assess potential risk factors early.

### Legal and ethical issues

The wide adoption of ML practices has heightened the debate concerning the legislative issues surrounding the issue inclusive of privacy protection. Many developers in the ML field struggle with establishing the appropriate trade-off between privacy and utility of various algorithms. Privacy protection remains a prominent issue in the field of ML due to limits of anonymization, data existing longer than the human subject it originated from, and data being used beyond their initial imagined purpose. Various researchers have explored these topics. For example, Bellini et al. argued that the development is happening so rapidly to the point that the legislative domain is experiencing challenges keeping up (28). Nevertheless, the success of ML in lung surgery will significantly depend on the amount and presence of high-quality data.

### Limitations

This study has three main limitations. This factor affected the derived results since the researchers could have excluded articles in other languages that provided information relevant to the study. The second limitation of the study is a wide literature gap in the field. This conclusion arises from the derivation of only nineteen articles to include in the research. The final limitation stems from adopting the narrative review approach since the technique led to broad findings, making it hard to draw essential conclusions.

## Conclusions

The outcomes of this study indicate that the lung surgery section has attempted to integrate machine learning algorithms. However, the implementation rate is low, owing to the newness of the concept and the various challenges it encompasses. Also, this study reveals the absence of sufficient literature discussing the application of machine learning in lung surgery. This lack of supporting evidence presents challenges for surgeons since they do not have enough information to guarantee the successful integration of ML techniques in the lung surgery sector. Furthermore, this study reveals the importance of more research into the area. For instance, future researchers should focus on testing more machine learning algorithms and evaluating their performance during lung surgery. Through this, it will be easier for lung surgeons to take advantage of the opportunities presented by machine learning and deal with the subsequent challenges.
